# Prenatal, Perinatal, and Postnatal Factors in a Cohort of Very Preterm and Very Low Birth Weight Toddlers with Suspected Autism Spectrum Disorder

**DOI:** 10.1007/s10803-025-06897-7

**Published:** 2025-06-04

**Authors:** Marta Marín Soro, Laura Gisbert Gustemps, Héctor Boix Alonso, Sergi Martínez-Maldonado, Ricard Coronado Contreras

**Affiliations:** 1 Institute of Neuroscience, Autonomous University of Barcelona, Barcelona, Spain; 2 Vall d’Hebron Hospital in Barcelona, Barcelona, Spain; 3 Autonomous University of Barcelona, Barcelona, Spain; 4 Quirón Hospital in Barcelona, Barcelona, Spain; 5 Open University of Catalonia, Barcelona, Spain; 6 General Hospital of Granollers / University Hospital of Mollet, Barcelona, Spain

**Keywords:** Associated factors, Autism spectrum disorder, Very preterm population, Prospective longitudinal study

## Abstract

There is growing interest in the identification of prenatal, perinatal, and postnatal factors that correlate with autism spectrum disorders (ASD) and these have been extensively studied in the general population. This study aimed to investigate the relationship between these factors and the elevated likelihood of being diagnosed with ASD in the very preterm and very low birth weight population. Conducted as a prospective longitudinal study, this research monitored 133 neonates born very preterm (less than 32 weeks of gestation) or weighing less than 1,500 g at birth from birth until 2 years of age. Having a mother born abroad, low gestational age, bronchopulmonary dysplasia, hearing loss, longer NICU stay and low Apgar score of 10 min were associated with an increase in suspected ASD. Conversely, cesarean delivery, and full-dose corticosteroid maturation were associated with a lower incidence of ASD. Some factors associated with ASD in the very preterm population may differ from those found in the general population. Large-scale studies with longitudinal datasets are warranted.

Autism spectrum disorders are neurological development disorders characterized, according to the Diagnostic and Statistical Manual of Mental Disorders (American Psychiatric Association, 2022) by deficits in communication and social interaction, and restrictive and repetitive behavior patterns. The prevalence of ASD has been estimated at 2.7% in the general population (Maenner et al., 2023), 1.23% in Catalonia (Pérez-Crespo et al., 2019), positioning it as a significant public health challenge. Although its etiology remains unknown, a meta‐analysis reported that 64–91% of autism could be explained by genetic factors while 7–35% is likely the result of shared environmental effects (Tick et al., 2016). The most plausible hypothesis suggests that an interaction between environmental and genetic factors could increase the probability of being diagnosed with autism. Previous work identified prematurity and low birth weight as factors correlated with autism. Thus, the prevalence of ASD significantly increases, reaching up to 6% in the premature population (< 37 weeks) (Laverty et al., 2021).

There is growing interest in finding environmental and modifiable predictive factors for ASD and this has been extensively studied in the general population, identifying some prenatal, perinatal, and postnatal factors associated with a diagnosis of ASD. Factors such as advanced parental age, gestational diabetes, maternal hypertension, assisted fertility, bleeding, and maternal birth abroad have been recognized as prenatal risk factors (Cogley et al., 2021; Gardener et al., 2009; Wang et al., 2017). Low gestational age, cesarean delivery, multiple births, complications during delivery, non-cephalic presentation, and preeclampsia are considered as perinatal risk factors (Gardener et al., 2011; Hisle-Gorman et al., 2018; Wang et al., 2017). Low birth weight, being small for gestational age, male sex, low Apgar score at 5 min, and brain abnormalities are associated with postnatal risk (Cogley et al., 2021; Gardener et al., 2011; Hisle-Gorman et al., 2018; Wang et al., 2017). However, research specifically focusing on very preterm infants remains scarce.

Due to the strong association between extreme prematurity and ASD, the Spanish Neonatology Society recommends applying a follow-up protocol for children up to 32 weeks of gestation and/or up to a birth weight of 1,500 g (Pallás Alonso et al., 2018). A previous study has demonstrated that this protocol is useful in significantly reducing the age of ASD detection in this population (Marín Soro et al., 2024). The protocol recommends specialized ASD screening with the Modified Checklist for Autism in Toddlers (M-CHAT) (Robins et al., 2014) for all premature children between 18 and 24 months of age and, if positive, performing a formal assessment with the Autism Diagnostic Observation Schedule (ADOS-2) (Lord et al., 2012). This follow-up requires that our study is of prospective longitudinal design which, unlike retrospective studies, allows for real-time, early data collection. The aim of this study is to examine the associations between prenatal, perinatal, and postnatal factors in a sample of very preterm toddlers with suspected ASD, and to explore whether these associations differ from those observed in the general population.

## Methods

### Study Design and Participants

This prospective longitudinal study implemented a Spanish follow-up protocol in very preterm children from birth until the age of two years. Between 2019 and 2021, neonates admitted to the Neonatal Intensive Care Unit (NICU) of a tertiary hospital in Catalonia (Spain) were recruited. Catalonia is a region with more than 8 million inhabitants, and the hospital where the study was conducted is the largest in the region, serving as a national reference center for neonatal care. Inclusion criteria were: (a) less than 32 weeks of gestation or (b) weight less than 1,500 g at birth. Children with severe motor impairment were excluded to avoid potential interference with the performance of ASD screening tools (Luyster et al., 2011; Shuster et al., 2023). The study received approval from the Clinical Research Ethics Committee of the Hospital in October, 2019.

Prenatal, perinatal, and postnatal data were collected during the neonate’s stay in the NICU. These data were later supplemented with the discharge report issued by the hospital and recorded in a comprehensive database. At 24 months of corrected age, M-CHAT-R/F (Robins et al., 2014) was administered to the parents or primary caregivers. Children who screened positive were formally assessed with the ADOS-2 (Lord et al., 2012) by a researcher clinically certified in ADOS with extensive clinical experience. The sessions were video-recorded with prior authorization from the families, and scores for each case were obtained. A cut-off point for ASD was established and a written report with the results and intervention recommendations was given to the parents. Informed consent was individually obtained from all study participants.

### Definition of Study Variables

The database includes records provided by medical service professionals and children’s family members.

The International Classification of Diseases, 10th Revision (ICD-10) identified prenatal diagnoses in mothers’ inpatient records. The discharge report described the mother’s age, use of assisted reproductive techniques and chorioamnionitis, an infection of the fetal membranes. Other sociodemographic variables, such as parents’ country of origin, parents’ education, father’s age, maternal substance use (legal and illegal drugs) and mental illness were collected through semi-structured interviews with the parents. Maternal and paternal age was categorized as ≥ 35 years.

Perinatal conditions were identified by ICD-10 codes in maternal and infant medical records, including maternal preeclampsia, a condition marked by elevated blood pressure and proteinuria after 20 weeks of pregnancy. Gestational age at birth was based on maternal reports of the last menstrual period and categorized in weeks and days. Birth weight, the season of birth, non-cephalic presentation, type of delivery as cesarean, arterial and venous pH values, corticosteroid maturation and sulfate neuroprotection were recorded from the hospital discharge report. Arterial and venous pH values were categorized as less than 7.28 and 7.20, respectively, as these thresholds indicate metabolic acidemia associated with perinatal hypoxia. Values below these cut-offs have been linked to an increased risk of neonatal complications, including neurodevelopmental impairment and adverse long-term outcomes. Corticosteroid maturation treatments are medical interventions administered to pregnant women at risk of preterm birth to accelerate the development of the fetal organs. In this study, the variable was recorded as “completed” if the full course of 2 doses was administered, and “incomplete” if only 1 dose was given. Sulfate neuroprotection refers to the administration of sulfate compounds to pregnant women at risk of preterm delivery to protect the developing fetal brain.

Neonatal conditions were identified and categorized by ICD-10 codes in infants’ medical records during admission to the NICU, including retinopathy of prematurity (ROP) categorized as grade ≥ 2, intraventricular hemorrhage (IVH) in grade 2 or higher and bronchopulmonary dysplasia (BPD). ROP is an abnormal development of retinal blood vessels and their grading system is typically used in clinical settings to describe severity. IVH refers to bleeding into the brain’s ventricular system, which contains cerebrospinal fluid, and its classification is based on the ICD-10 system. Both ROP and IVH were categorized starting from grade 2, as this threshold has been associated with a higher risk of long-term neurodevelopmental impairments, making it a clinically relevant distinction. BPD originating in the perinatal period involves chronic lung inflammation. Sensorineural hearing loss, a type of hearing impairment, was detected through auditory brainstem response (ABR) testing within the first few months of life. Sex, length, head circumference, Apgar scores at 5 and 10 min categorized as < 7 and the duration of NICU stay in days were recorded from the discharge report. To determine growth according to gestational age at birth, subjects were classified following Carrascosa’s charts (Carrascosa et al., 2004) into three categories: small (below the 10th percentile), appropriate (between the 10th and 90th percentiles), and large (above the 90th percentile).

The study cohort was prospectively followed from birth to 2 years for the earliest recorded detection of ASD, which was based on screening evaluations and specific assessments.

The Modified Checklist for Autism with Follow-up Interview (M-CHAT-R/F) (Robins et al., 2014) is a screening questionnaire for autism applicable from 16 to 30 months of age. It consists of 20 items, and the interview is considered screen positive if the child fails any two items. The follow-up interview significantly enhances the predictive values for ASD (Guy et al., 2015), with a pooled sensitivity of 83% and a pooled specificity of 94% (Wieckowski et al., 2023).

Autism Diagnostic Observation Schedule 2 (ADOS-2) (Lord et al., 2012) is a structured and standardized assessment tool for autism that involves direct observation of the evaluated subjects’ behavior while they engage in various activities. It includes a Toddler Module, tailored for assessing children between 12 and 30 months. It gathers 41 scores based on language, social communication, and relevant behaviors. For the clinical diagnosis of autism, the most accurate cut-off point is 12 points for non-speaking children and 10 points for children with some words (Hong et al., 2021). The area under the curve (AUC) of these cutoff points is 0.92 and 0.96 respectively, indicating excellent diagnostic validity. We define children with suspected ASD as those whose scores are equal to or above these cut-off points.

### Statistical Analysis

In our study, we conducted statistical analyses using R version 4.2.2 (R Core Team, 2022). We assessed the impact of various prenatal, perinatal, and neonatal factors on ASD screening outcomes through logistic regression, calculating relative risks (RRs) for binary variables and odds ratios (ORs) for continuous or non-binary categorical variables, each with corresponding 95% confidence intervals and *p*-values.

For the unadjusted models, chi-square was applied for binary outcomes or Fisher’s exact tests were used when any cell value in the contingency table was lower than 5. For continuous or non-binary categorical predictors, the Wald test was used. To account for gestational age as a confounding variable, it was included as a covariate in the adjusted logistic regression models. While birth weight is also a significant factor related to prematurity, gestational age and birth weight were found to be highly correlated in our data (*r* = 0.72). Therefore, to mitigate potential multicollinearity issues and ensure model parsimony in our exploratory analysis with a limited sample size, we opted to primarily adjust for gestational age in our models.

Our data were nearly complete, with less than 2.5% of missing values. To address them, we employed imputation strategies tailored to each variable type. Median values were used for missing demographic data, particularly for single-parent cases. Linear regression models, informed by correlated variables such as gestational age and birth weight, were used for missing anthropometric and Apgar scores. Similarly, regression models, considering relevant clinical variables, were utilized for imputing missing pH values, ensuring the integrity and completeness of our analysis. Further information is provided in the supplementary material.

Various statistical methods, including G-Power 3.1 (Faul et al., 2009) and the Wilcoxon rank-sum test (R Core Team, 2022), were used to determine the sample size, confirming its adequacy for statistical findings.

## Results

A total of 133 neonates were included. From the screening conducted with the M-CHAT-R/F at 24 months of age, 80 (60.15%) were negative and 53 (39.85%) were positive. Of the 53 cases identified as positive, the evaluation with the ADOS-2 Toddler Module was completed in 50 (94.34%), with 35 administered to preverbal children or those with few words, and 15 to infants who regularly used some words. A total of 3 losses were recorded, all male children. A total of 24 subjects met the ADOS-2 most accurate cut-off point for ASD diagnosis, defined as a score of ≥ 12 for non-speaking children and ≥ 10 for children with some words, representing 18.46% of the entire sample.

In our study, the only prenatal factor significantly associated with suspected ASD was having a mother who was born abroad (Table 1).

**Table 1:** Bivariate associations between prenatal factors and suspected ASD

Associated factors	Sample size (*N* = 133)	Positive screening (M-CHAT)		ASD cutoff point (ADOS-2)	
		Summary effect estimate (95% CI)	*P* value	Summary effect estimate (95% CI)	*P* value
Mother born abroad	67 (50,38%)	RR = 1.42, 95% CI 0.97–2.09	0.06	RR = 2.23, 95% CI 1.09–4.54	0.01
Mother’s education: up to secondary	44 (33.10%)	RR = 1.37, 95% CI 1.04–1.81	0.01	RR = 1.20, 95% CI 0.83–1.72	0.28
Father’s education: up to secondary	50 (*n* = 131) (38.17%)	RR = 1.25, 95% CI 0.93–1.67	0.13	RR = 1.24, 95% CI 0.82–1.88	0.26
Mother age ≥ 35	55 (41.35%)	RR = 0.95, 95% CI 0.71–1.27	0.74	RR = 1, 95% CI 0.69–1.46	0.99
Father age ≥ 35	74 (*n* = 131) (55.64%)	RR = 1.55, 95% CI 0.99–2.42	0.04	RR = 1.29, 95% CI 0.71–2.34	0.38
Mother and father age ≥ 35	46 (*n* = 131) (34.59%)	RR = 1.03, 95% CI 0.80–1.33	0.80	RR = 1.06, 95% CI 0.75–1.48	0.74
Mother substance misuse	18 (13.53%)	(RR = 0.99, 95% CI 0.87–1.14)	0.93	RR = 0.88, 95% CI 0.78–0.99)	0.19
Maternal disease	65 (48,90%)	RR = 0.89, 95% CI 0.64–1.24	0.50	RR = 0.74, 95% CI 0.52–1.04)	0.12
ART	38 (28.60%)	RR = 0.99, 95% CI 0.80–1.24	0.96	(RR = 1.01, 95% CI 0.76–1.34)	0.93
Chorioamnionitis	38 (28.60%)	RR = 1.04, 95% CI 0.83–1.30	0.74	RR = 1.10, 95% CI 0.81–1.50	0.49

ART = assisted reproductive technologies

As for perinatal factors, both low gestational age, categorized by days and weeks, and a weight of less than 750 g were associated with an increased risk of suspected ASD. Cesarean delivery and a complete course of corticosteroid maturation were associated with a lower probability of ASD. The presence of preeclampsia showed a trend towards significance (Table 2).

**Table 2:** Bivariate associations between perinatal factors and suspected ASD

Associated perinatal factors	Sample size (*N* = 133)	Positive screening (M-CHAT)		ASD cutoff point (ADOS-2)	
		Summary effect estimate (95% CI)	*P* value	Summary effect estimate (95% CI)	*P* value
Gestational age (weeks)	28.87 (2.83)	(OR = 0.87, 95% CI 0.76–0.99)	0.03	OR = 0.83, 95% CI 0.70–0.98	0.03
Gestational age (days)	204.96 (19.58)	OR = 0.98, 95% CI 0.96-1	0.03	OR = 0.97, 95% CI 0.95–0.99	0.02
Birth weight (kg)	1139.89 (343.77)	(OR = 0.99, 95% CI 0.99-1.00)	0.06	OR = 0.99, 95% CI 0.99-1.00	0.13
Extremely Low Birth weight (< 750)	19 (14.29%)	(RR = 1.23, 95% CI 1.04–1.45)	0.006	RR = 1.25, 95% CI 0.96–1.63	0.03
Spring birth (ref: fall)	21 (15.79%)	OR = 0.58, 95% CI 0.19–1.73	0.33	OR = 0.97, 95% CI 0.26–3.69	0.97
Winter birth (ref: fall)	35 (26.32%)	OR = 0.87, 95% CI 0.35–2.15	0.76	OR = 0.92, 95% CI 0.28–2.97	0.88
Summer birth (ref: fall)	36 (27.07%)	OR = 0.58, 95% CI 0.23–1.46	0.25	OR = 0.85, 95% CI 0.26–2.75	0.79
Preeclampsia	32 (24,06%)	RR = 0.82, 95% CI 0.69–0.99	0.049	RR = 0.78, 95% CI 0.66–0.93	0.06
Cesarean Delivery	111 (83.46%)	RR = 0.55, 95% CI 0.26–1.19	0.012	RR = 0.33, 95% CI 0.16–0.68	< 0.001
Non-cephalic Presentation	59 (44.36%)	RR = 0.95, 95% CI 0.78–1.16	0.64	RR = 1.08, 95% CI 0.82–1.42	0.57
Twins or multiple birth	54 (40.60%)	RR = 1.35, 95% CI 0.98–1.85	0.048	RR = 1.15, 95% CI 0.77–1.71	0.46
Arterial ph < 728	50 (46,30%) (*n* = 108)	RR = 0.91, 95% CI 0.64–1.29	0.59	RR = 0.96, 95% CI 0.62–1.50	0.87
Venous ph < 720	9 (8.74%) (*n* = 103)	RR = 0.93, 95% CI 0.83–1.04	0.30	RR = 0.96, 95% CI 0.84–1.09	> 0.99
Complete corticosteroid maturation	103 (77.44%)	RR = 0.58, 95% CI 0.31–1.09	0.09	RR = 0.45, 95% CI 0.24–0.84	0.02
Sulfate neuroprotection	108 (83.08%) (*n* = 130)	RR = 2.34, 95% CI 0.92–5.95	0.059	RR = 1.05, 95% CI 0.39–2.82	> 0.99

Finally, significant postnatal factors were bronchopulmonary dysplasia, hearing loss, longer NICU stay and low 10-minute Apgar scores. The presence of ROP, short length and cranial circumference at birth showed weak evidence (Table 3).

**Table 3:** Bivariate associations between neonatal factors and suspected ASD

Associated neonatal factors	Sample size (*N* = 133)	Positive screening (M-CHAT)		ASD cutoff point (ADOS-2)	
		Summary effect estimate (95% CI)	*P* value	Summary effect estimate (95% CI)	*P* value
Sex: male	68 (51.13%)	RR = 0.93, 95% CI 0.66–1.32	0.70	RR = 1.25, 95% CI 0.75–2.07	0.37
Large for gestational age	3 (2.26%)	RR = 0.99, 95% CI 0.94–1.05	> 0.99	RR = 0.97, 95% CI 0.94-1.00	> 0.99
Small for gestational age	41 (30.83%)	RR = 1.03, 95% CI 0.82–1.30	0.80	RR = 0.83, 95% CI 0.65–1.07	0.24
Length (cm)	36.38 (4,15)	OR = 0.91, 95% CI 0.83–0.99	0.03	OR = 0.91, 95% CI 0.82–1.02	0.10
Head circumference (cm)	26.23 (2.88)	OR = 0.88, 95% CI 0.78-1.00	0.06	OR = 0.85, 95% CI 0.71–0.999	0.06
5-min Apgar score < 7	33 (25.19%) (*n* = 131)	RR = 1.07, 95% CI 0.87–1.32	0.50	RR = 1.25, 95% CI 0.90–1.73	0.12
10-min Apgar score < 7	9 (7.63%) (*n* = 118)	RR = 1.13, 95% CI 1.00-1.28	0.03	RR = 1.31, 95% CI 1.02–1.67	< 0.001
IVH ≥ 2	15 (11.28%)	RR = 1.07, 95% CI 0.94–1.23	0.26	RR = 1.14, 95% CI 0.92–1.42	0.11
BPD	37 (27.82%)	RR = 1.53, 95% CI 1.18–1.99	< 0.001	RR = 1.69, 95% CI 1.08–2.64	< 0.001
ROP ≥ 2	26 (19.55%)	RR = 1.20, 95% CI 0.99–1.46	0.04	RR = 1.25, 95% CI 0.93–1.67	0.07
Sensorineural hearing loss	14 (10.53%)	RR = 1.13, 95% CI 0.99–1.29	0.048	RR = 1.23, 95% CI 0.97–1.56	0.01
Admission duration (days)	69.55 (36.02)	OR = 1.02, 95% CI 1.01–1.03	< 0.001	OR = 1.03, 95% CI 1.02–1.05	< 0.001

IVH = intraventricular hemorrhage; BPD = bronchopulmonary dysplasia; ROP = retinopathy of prematurity; admission duration = premature infant stays in NICU

Additionally, some variables that were not found to be significantly associated with suspected ASD did show a significant relationship with a positive screening through the M-CHAT. These included paternal age of 35 years or older, maternal education up to secondary, multiple gestation and grade 2 or more of ROP.

## Discussion

This study was designed to investigate the association between specific factors and high probability of ASD diagnosis in a cohort of very preterm children. The results suggested, as hypothesized, that certain prenatal, perinatal, and postnatal factors might be associated with the likelihood of ASD. In accordance with previous findings, confirmed by this study, preterm children require thorough follow-up due to the high probability of neurodevelopmental deficits.

The only prenatal factor associated with suspected ASD identified in our study was maternal foreign birth, referring to mothers born outside of Spain. This is consistent with some prior findings (Becerra et al., 2014; Gardener et al., 2009), although other studies have reported contrasting results (Fairthorne et al., 2017). Regional characteristics may account for the discrepancies in outcomes. Susceptibility to relatively benign infections and the stress associated with residing in a new country have been proposed as potential explanations (Becerra et al., 2014; Gardener et al., 2009). Other factors commonly linked to ASD in the general population, such as assisted reproductive techniques and advanced paternal age, did not show any association in our study.

The results suggest, as hypothesized, that low gestational age was the most relevant perinatal variable associated with ASD in our study. The younger the gestational age, whether assessed in days or weeks, the higher the likelihood of suspected ASD. A recent meta-analysis found that premature infants are 3.3 times more likely to develop the disorder, although it did not find gestational age to be significantly related to autistic traits (Laverty et al., 2021). Nonetheless, several studies have consistently reported an association between lower gestational age and increased risk of ASD (Guo et al., 2022; Hwang et al., 2013; Johnson et al., 2010; Joseph et al., 2017a, b Kuzniewicz et al., 2014). The heterogeneity found may be due to differences in the categorization of the gestational age variable (Cogley et al., 2021). It has been suggested that the possible relationship is due to the vulnerability of the immature brain, the lack of neuroprotective factors or physiological instability (Joseph et al., 2017b). Another factor widely related to ASD is low birth weight (Gardener et al., 2011; Guo et al., 2022; Ma et al., 2022; Pyhälä et al., 2014; Wang et al., 2017). However, our results only indicated a weak relationship between these variables, except when the birth weight was extremely low (< 750 g). This suggests that in the premature population, gestational age may be a more determining factor than birth weight for the development of ASD.

Additionally, according to our results, cesarean delivery was associated with low probability of ASD diagnosis. This contrasts with findings from previous studies. Cesarean delivery has been associated with a higher likelihood of ASD in a comprehensive meta-analysis (Curran et al., 2015), although another study found no significant relationship in the premature population (Yip et al., 2016). Cesarean delivery is usually indicated when there are risks associated with prematurity. Our findings differ from other publications and likely require a larger study to evaluate the role of cesarean section, taking into account the indication for the cesarean section, the clinical status of the fetus and the mother, as well as the subsequent stabilization of the newborn. Another factor associated with a lower probability of ASD identified in our study was the complete administration of corticosteroids for fetal lung maturation. Corticosteroid administration is considered an effective means of reducing perinatal mortality and morbidity, as well as the risk of developmental delay (McGoldrick et al., 2020), although we are not aware of any previous studies linking it to ASD.

Preeclampsia scores trend towards significance as a protective factor for ASD, contrary to some studies suggesting it could be a risk factor for ASD (Jenabi et al., 2019; Maher et al., 2020; Wang et al., 2017). However, these studies involve a general population, without considering gestational age. One explanation could be that preeclampsia contributes to the development of ASD due to its association with prematurity, as the presence of the condition often leads to premature birth. In our study, the population consists exclusively of very premature infants, and if preeclampsia is the reason for prematurity, perhaps other variables more harmful to the fetus are not present.

During the neonatal period, our findings indicated an association between autism and a series of complications that commonly occur in premature infants. Specifically, bronchopulmonary dysplasia (BPD) and hearing loss were identified. These relationships remained significant even when controlling for gestational age. Previous studies have found a link between BPD and ASD (Hack et al., 2009) or associated characteristics (Brumbaugh et al., 2018; Sriram et al., 2018). Additionally, the relationship between hearing impairment and ASD has been extensively explored. Several studies have found a higher prevalence of hearing impairment in the ASD population (Demopoulos & Lewine, 2016; Ocak et al., 2018; Rydzewska et al., 2019; Ting et al., 2023), although a previous literature review did not find conclusive data (Beers et al., 2014). Structural and functional abnormalities have been found in the auditory brainstem in individuals with ASD, which could explain this relationship (Smith et al., 2019). Another associated factor identified in our study is the duration of stay in the NICU. This variable has previously been associated with ASD (Subedi et al., 2017; Winkler-Schwartz et al., 2014). Exposure to stressors associated with the NICU can alter the brain both functionally and structurally (Smith et al., 2011), which could explain the emergence of neurodevelopmental disorders such as ASD. Lastly, an Apgar score at 10 min lower than 7 has also been identified as an associated factor for ASD. This relationship remains significant when controlling for gestational age. Most previous studies agree that a low Apgar score at 5 min is associated with the presence of ASD (Gardener et al., 2011; Getahun et al., 2017; Modabbernia et al., 2019; Wang et al., 2017). It has been postulated that neonatal vitality and ASD share a common genetic background, or that the role of environmental factors such as birth complications in the development of ASD could be possible explanations for this relationship (Modabbernia et al., 2019). The variables that showed a trend towards significance were low birth length, head circumference, and ROP grade 2 or higher. Regarding ROP, it has also been associated with ASD, suggesting a common pathway between retinal issues and neurodevelopmental disorders.

Our results suggest that some factors associated with ASD in the preterm population could differ from those found in the general population. While gestational age and having a mother born abroad or hearing loss remain associated, cesarean delivery or complete maturation with corticosteroids could reduce the probability of ASD diagnosis. Certain aspects related to prematurity, such as ROP, bronchopulmonary dysplasia, low Apgar score or length of stay in the NICU, would be associated factors even when controlling for gestational age as a confounding variable.

Some variables in our study were associated with an increased likelihood of obtaining a positive screening on the M-CHAT, but not with a higher probability of subsequent ASD; specifically, paternal age equal to or greater than 35 years, mother’s education up to secondary, multiple gestation, preeclampsia, short birth length and grade 2 ROP. Several previous studies indicated that those registering false positives on the M-CHAT are at risk of cognitive, sensory, and behavioral problems (Luyster et al., 2011; Moore et al., 2012), so these factors are likely to pose a higher risk of other disorders or difficulties.

The rate of positive M-CHAT-R/F screens in our study is higher than that reported by other recent cohorts of premature infants (Shuster et al., 2023). This difference may be partially explained by variations in exclusion criteria or by cultural differences influencing parental reporting and interpretation of developmental behaviors within our population. Additionally, the higher rates of suspected ASD cases observed in our cohort compared to larger population-based studies (Crump et al., 2021; Joseph et al., 2017b) likely result from our early assessment at 24 months using the ADOS-2 Toddler Module, whereas those studies relied on formal clinical diagnoses at older ages.

Some limitations of the study need to be considered when interpreting the results. Firstly, the sample size, as although the sample size calculation indicated that the number of subjects was adequate, larger samples would enhance statistical power and representativeness. Additionally, a limited number of cases hinders discussion of causal relationships and, consequently, risk factors, but it does allow for establishing positive and negative associations between diverse variables and laying a foundation for future research. Moreover, standardized cognitive, language, or motor scores were not included in the analysis. Given the neurodevelopmental vulnerability of preterm children, these scores could provide valuable insights into their developmental trajectories and potential confounding factors. Finally, it should be noted that the detection of ASD is based on a cut-off point and, as indicated by the Spanish protocol (Pallás Alonso et al., 2018), follow-up should continue until adolescence to confirm the diagnosis.

Although previous prospective studies (Joseph et al., 2017b; Shuster et al., 2023), have explored risk factors for ASD in extremely preterm infants, research on this topic remains limited, particularly regarding early identification and prospective follow-up in clinical settings. This design allows for earlier detection of warning signs, which is essential for prevention and targeted intervention programs. Lastly, the administration of the M-CHAT-R/F follow-up interview and the ADOS-2 was carried out by the same rater in all cases. While having a single examiner may limit inter-rater reliability and introduce potential biases related to examiner expectations or interpretation, it also minimizes discrepancies in scoring due to subjective differences between multiple raters. Additionally, using a single evaluator enhances family adherence to the study and facilitates coordination with other professionals involved in the child’s care.

The high probability of neurodevelopmental disorders such as ASD in the very preterm population underscores the need for future prospective longitudinal studies with larger samples aimed at shedding light on these findings and elucidating modifiable associated factors in early childhood. This follow-up promotes early detection and intervention, thus improving the quality of life for individuals with ASD.
